# Human adipose mesenchymal stem cells modulate myeloid cells toward an anti-inflammatory and reparative phenotype: role of IL-6 and PGE2

**DOI:** 10.1186/s13287-020-01975-2

**Published:** 2020-11-02

**Authors:** Maitane Ortiz-Virumbrales, Ramón Menta, Laura M. Pérez, Ornella Lucchesi, Pablo Mancheño-Corvo, Álvaro Avivar-Valderas, Itziar Palacios, Angel Herrero-Mendez, Wilfried Dalemans, Olga de la Rosa, Eleuterio Lombardo

**Affiliations:** 1 Takeda Madrid, Cell Therapy Technology Center, Tres Cantos, Spain; 2 TiGenix NV, Leuven, Belgium

**Keywords:** Adipose-derived mesenchymal stem cells, Anti-inflammatory, Monocytes, Macrophages, Dendritic cells, Interleukin 6, Prostaglandin E2

## Abstract

**Background:**

Mesenchymal stem cells (MSCs) activate the endogenous immune regulatory system, inducing a therapeutic effect in recipients. MSCs have demonstrated the ability to modulate the differentiation of myeloid cells toward a phagocytic and anti-inflammatory profile. Allogeneic, adipose-derived MSCs (ASCs) have been investigated for the management of complex perianal fistula, with darvadstrocel being the first ASC therapy approved in Europe in March 2018. Additionally, ASCs are being explored as a potential treatment in other indications. Yet, despite these clinical advances, their mechanism of action is only partially understood.

**Methods:**

Freshly isolated human monocytes from the peripheral blood were differentiated in vitro toward M0 non-polarized macrophages (Mphs), M1 pro-inflammatory Mphs, M2 anti-inflammatory Mphs, or mature dendritic cells (mDCs) in the presence or absence of ASCs, in non-contact conditions. The phenotype and function of the differentiated myeloid populations were determined by flow cytometry, and their secretome was analyzed by OLINK technology. We also investigated the capacity of ASCs to modulate the phenotype and function of terminally differentiated M1 Mphs. The role of soluble factors interleukin (IL)-6 and prostaglandin E2 (PGE2) on the ability of ASCs to modulate myeloid cells was assessed using neutralization assays, CRISPR/Cas9 knock-down of cyclooxygenase 2 (COX-2), and ASC-conditioned medium assays using pro-inflammatory stimulus.

**Results:**

Co-culture of monocytes in the presence of ASCs resulted in the polarization of Mphs and mDCs toward an anti-inflammatory and phagocytic phenotype. This was characterized by an increase in phagocytic receptors on the cell surface of Mphs (M0, M1, and M2) and mDCs, as well as modulation of chemokine receptors and reduced expression of pro-inflammatory, co-stimulatory molecules. ASCs also modulated the secretome of Mphs and mDCs, demonstrated by reduced expression of pro-inflammatory factors and increased expression of anti-inflammatory and reparative factors. Chemical inhibition of PGE2 with indomethacin abolished this modulatory effect, whereas treatment with a neutralizing anti-IL-6 antibody resulted in a partial abolishment. The knock-down of COX-2 in ASCs and the use of IL-1β-activated ASC-conditioned media confirmed the key role of PGE2 in ASC-mediated myeloid modulation. In our in vitro experimental settings, ASCs failed to modulate the phenotype and function of terminally polarized M1 Mphs.

**Conclusions:**

The results demonstrate that ASCs are able to modulate the in vitro differentiation of myeloid cells toward an anti-inflammatory and reparative profile. This modulatory effect was mediated mainly by PGE2 and, to a lesser extent, IL-6.

## Background

Circulating monocytes originate from the bone marrow and migrate to tissues where they encounter various soluble factors and cytokines, resulting in their differentiation into macrophages (Mphs) and dendritic cells (DCs) [1]. Macrophage colony-stimulating factor (M-CSF) and granulocyte-macrophage colony-stimulating factor (GM-CSF) are the main factors driving the differentiation of monocytes into Mphs during both homeostatic and inflammatory conditions [2]. Polarization of Mphs toward distinct subsets with either an anti- or pro-inflammatory profile is dependent upon additional signals that they encounter during the differentiation process, such as pro/anti-inflammatory cytokines [1]. Activation of type 1 T helper (Th1) cells and natural killer cells results in the secretion of interferon (IFN)-γ. This process, combined with activation of Toll-like receptors (TLRs) and the secretion of tumor necrosis factor (TNF), leads to the differentiation of Mphs into classically activated, pro-inflammatory M1 Mphs [3]. In contrast, interleukin (IL)-10, produced by regulatory T cells, results in the induction of type 2 T helper (Th2) cells, which secrete IL-4 and IL-13, promoting the development of activated, anti-inflammatory M2 Mphs [3]. The presence of GM-CSF and IL-4 promotes differentiation of monocytes into immature DCs (iDCs), which can further differentiate into mature DCs (mDCs) upon activation with lipopolysaccharide (LPS) to initiate the adaptive immune response [4]. In the case of persistent infection or inflammation, myeloid cells acquire a pro-inflammatory phenotype, which subsequently perpetuates the inflammatory process [1]. This exacerbation of inflammation underlies several inflammatory diseases, such as Crohn’s disease, ulcerative colitis, rheumatoid arthritis, and sepsis [5, 6]. Therefore, modulating the function of these myeloid populations can provide the ability to promote the resolution of inflammatory conditions.

Mesenchymal stem cells (MSCs) are multipotent stromal cells that exist in almost all tissues, where they have been described as having a perivascular localization [7]. Besides being a source of reserve cells with the ability to differentiate into a range of cell types, MSCs also demonstrate immunomodulatory, anti-fibrotic, anti-apoptotic, and pro-angiogenic properties, and their therapeutic potential has been reported in several studies [8–10]. MSCs possess the ability to modulate the phenotype and function of a range of immune cells through direct cell-to-cell interactions, immunomodulatory factors, and secretion of growth factors [11, 12]. MSCs have demonstrated a therapeutic effect in vivo in several inflammatory and autoimmune-related conditions. This effect was mediated through their modulation of immune cells toward regulatory phenotypes, such as regulatory T cells and immunomodulatory Mphs [13, 14]. The key role that Mphs play in the ability of MSCs to exert their modulatory effect was demonstrated in vivo when depletion of host Mphs abolished the therapeutic effect of MSCs [14]. However, the full mechanism of action of MSCs needs further investigation.

In recent years, the ability of MSCs to modulate monocyte and Mph function toward an anti-inflammatory phenotype has been investigated, both in vitro and in vivo [15–17]. Data demonstrate that MSCs are able to downregulate the activation state of myeloid populations, enhance the phagocytic function of Mphs and DCs, and modulate their secretomes toward an anti-inflammatory profile [18–21]. MSCs have also shown the ability to modulate the differentiation of monocytes to mDCs in vitro, generating a cluster of differentiation (CD) 14^+^ CD1a^−^ population with enhanced regulatory properties [18, 22–24].

Although MSCs from different sources share many characteristics, they can demonstrate significant variability in their immunomodulatory properties depending on their source [25]. The majority of the published data describing the modulatory effects of MSCs on Mphs and DCs refer to bone marrow MSCs or umbilical cord MSCs, but the current understanding of the interactions between adipose-derived MSCs (ASCs) and myeloid cells, which is key for their applicability in vivo, is limited and yet to be studied comprehensively.

Here, we aimed to characterize the modulatory effect of ASCs on human myeloid cells in vitro and explore the mechanism by which they exert this modulation.

## Methods

### Isolation and culture of human ASC

Freshly isolated human adipose aspirates were washed twice with phosphate-buffered saline (PBS) and subsequently digested with 0.075% collagenase (type I, Invitrogen, Carlsbad, CA, USA). The digested sample was then washed with 10% fetal bovine serum (FBS) and treated with 160 mM ammonium chloride in order to eliminate any residual erythrocytes. FBS from Australian origin was gamma-irradiated and heat-inactivated, provided by Thermo Scientific. The FBS-washed ASCs were suspended in a culture medium (Dulbecco’s modified Eagle’s medium with 10% FBS). The cells were seeded [2, 3] (× 1000 cells/cm^2^) in tissue culture flasks and expanded (37 °C, 5% carbon dioxide [CO_2_]) with a change of culture medium every 3–4 days. When the cells had reached 90% confluence, they were transferred to a new culture flask. Further expansion protocol has been previously published [26]. ASCs fulfilled the International Society for Cellular Therapy (ISCT) criteria for MSCs and were thoroughly checked for viability, population doublings, morphology, potency, identity, purity, sterility, and genetic stability, among other quality controls [27]. ASCs were obtained from healthy donors who had provided informed consent under the auspices of the appropriate research and ethics committees [28]. Three different ASC donors were used in this study.

### Blood samples

Buffy coats were obtained from healthy volunteers after informed consent from the Transfusions Center at Comunidad de Madrid, Madrid, Spain. Approximately 50–60 mL of blood was diluted with PBS at room temperature and distributed into 50-mL tubes with the addition of Ficoll^®^ Paque Plus (15 mL at room temperature). The samples were centrifuged for 40 min (2000 rpm at 10 °C) without brake or acceleration. The white rings of peripheral blood mononuclear cells (PBMCs) were collected, washed once in 50 mL of cold PBS, and centrifuged for a further 15 min (1800 rpm at 10 °C) without acceleration or brake. Following the second wash in 50 mL of cold complete Roswell Park Memorial Institute (RPMI) (RPMIc; RPMI with 10% FBS, l-glutamine, and penicillin/streptomycin), the samples were centrifuged for 15 min (1500 rpm at 10 °C) with brake and acceleration. The final wash was carried out using 50 mL of cold RPMIc, and the resulting PBMCs were centrifuged for 15 min (1200 rpm at 10 °C) with brake and acceleration. PBMCs were then re-suspended in RPMIc and counted, followed by a second re-suspension at 100 million per milliliter in cold RPMIc with the addition of an equal volume of cold RPMIc supplemented with 10% dimethyl sulfoxide. Finally, PBMCs were frozen in liquid nitrogen in vials of 50 million cells. Buffy coats from seven different healthy donors were used for the initial phenotypic and functional characterization assays, and eight additional donors were used for mechanistic studies (IL-6, PGE2, COX-2 knock-out [KO], and conditioned media).

### Isolation of CD14^+^ CD16^-^ monocytes

Frozen vials of PBMCs were thawed and counted, and CD14^+^ CD16^−^ monocytes were isolated using the Dynabeads^®^ Untouched Human Monocytes kit (Dynal^®^ Thermo Fisher Scientific, Waltham, MA, USA), following the manufacturer’s instructions.

### Culture and differentiation of human monocytes

The isolated CD14^+^ CD16^−^ monocytes were plated in 5 mL of RPMIc at 1.5 million per 6-well (Falcon^®^, Corning Biosciences, Corning, NY, USA). The following factors were added for differentiation into non-polarized M0 Mphs or further polarization into M1 Mphs, M2 Mphs, or mDCs in monocultures. For M0 Mphs, 50 ng/mL M-CSF was added for 5–6 days. For M1 Mphs, 50 ng/mL M-CSF was added with the addition of 30 ng/mL IFN-γ (eBioscience, Thermo Fisher Scientific, Waltham, MA, USA) + 20 ng/mL *Escherichia coli* (*E. coli*) LPS (Millipore Sigma, St. Louis, MO, USA) at day 3 for 3 more days. For M2 Mphs, 50 ng/mL M-CSF + 20 ng/mL IL-4 + 20 ng/mL IL-13 were added for 6 days. For iDCs, 5 ng/mL GM-CSF + 10 ng/mL IL-4 were added for 5 days; maturation of DCs was induced at day 5 through the addition of 40 ng/mL LPS for 2 more days. Human recombinant M-CSF, GM-CSF, IL-4, and IL-13 were from PeproTech (Rocky Hill, NJ, USA). Cells were differentiated in tissue culture incubators (37 °C, 5% CO_2_).

### Co-culture experiments with ASC

Freshly isolated human CD14^+^ CD16^−^ monocytes were co-cultured with ASCs from donor 10 or donor 13 in polycarbonate 6-well transwells with a 0.4-μm pore size (Transwell^®^ Permeable Supports and Falcon^®^ plates [Corning Biosciences, Corning, NY, USA]). One hundred fifty thousand ASCs were plated on the transwell inserts in 1 mL of media, 16 h prior to co-culture. During co-culture, 1.5 million monocytes were placed at the bottom of the well in 4 mL of media. Differentiation was achieved using the same factors used in the differentiation of monocytes alone, and ASCs were kept in the transwell insert for the entire duration of the differentiation process. To investigate the ability of ASCs to revert polarization of M1 Mphs, monocytes were fully differentiated toward M1 Mphs in the absence of ASCs using M-CSF for 4 days and then adding IFN-γ and LPS for an additional 2 days. ASCs were then incorporated into a transwell co-culture setting as described above for 4 extra days. Cells were differentiated in tissue culture incubators (37 °C, 5% CO_2_).

### Phagocytosis assays

After differentiation in monoculture or co-culture in vitro, Mphs and mDCs were harvested using StemPro^®^ Accutase^®^ (Thermo Fisher Scientific, Waltham, MA, USA) for 20 min at 37 °C. The phagocytic potential of polarized Mph or mDC was assessed using pHrodo™ red-conjugated yeast and bacterial particles (Zymosan A, *E. coli*, or *Staphylococcus aureus* [*S. aureus*]; Life Technologies, Carlsbad, CA, USA) following the manufacturer’s instructions. These particles can detect the acid pH of the phagosome and fluoresce to indicate that phagocytosis is taking place. Fifty thousand cells were transferred to 96-well Ultra-Low Attachment U-bottom wells (Corning Biosciences, Corning, NY, USA), and cells were rested for 60 min in RPMIc. Lyophilized pHrodo™-conjugated particles were reconstituted in 1 mL of RPMIc per vial prior to use, and particles were sonicated for 5 min at 20% amplitude. pHrodo™ Zymosan or bacterial particles (50 μL) were added to each well, and the cells were incubated for 60 min (37 °C, 5% CO_2_). Afterwards, phagocytosis was stopped by placing the samples on ice, and cells were washed prior to fluorescence-activated cell sorting analysis using a LRSFortessa™ cytometer (BD Biosciences, Haryana, India). Negative controls for phagocytosis were cells without pHrodo™ reagent. The results were analyzed using the FlowJo™ software.

### Phenotypic characterization

After differentiation in monoculture or co-culture in vitro, Mphs and mDCs were harvested using StemPro^®^ Accutase^®^ (Thermo Fisher Scientific, Waltham, MA, USA) for 20 min at 37 °C, after supernatants were collected and frozen for future OLINK analysis. Cells were counted and then distributed into 96-well V-bottom plates for staining (Nunc™, Thermo Fisher Scientific, Waltham, MA, USA). Cells were incubated in blue magnetic-activated cell sorting (MACS) buffer with 1% human serum for 15 min on ice, in order to block Fcγ receptor-mediated unspecific antibody binding. Subsequently, cells were stained for 20 min on ice with suitable combinations of antibody mixes, isotype controls, or unstained controls. We used suitable combinations of anti-CD1a PE, anti-CD14 APC, anti-CD16 PE, anti-CD45 FITC, anti-CD64 PE, anti-CD68 PECy7, anti-CD80 FITC, anti-CD83 PE, anti-CD86 PE, anti-CD115 PE, anti-CD163 PE, anti-183 APC, anti-CD183 Alexa488, anti-CD185 Alexa488, anti-CD192 Alexa647, anti-CD193 PE, anti-CD197 FITCA, anti-CD206 PE, anti-CD209 FITC, anti-CX3CR1 PE, and anti-HLA DP DQ DR FITC. All antibodies and isotype controls were from BD Biosciences (Haryana, India), except anti-CD64 PE (Miltenyi Biotech, North Rhine-Westphalia, Germany). Cell viability was assessed by the addition of 7-aminoactinomycin D (7-AAD), and samples were analyzed using a LRSFortessa™ cytometer (BD Biosciences, Haryana, India). The results were analyzed using the FCS Express Software and FlowJo™. At the end of monocyte differentiation, both in the presence and absence of ASCs, the conditioned media (containing the secretomes [sets of secreted proteins] of differentiated Mphs and mDCs) were harvested for proteomic analysis.

### OLINK analysis

A proteomic analysis system based on the Proximity Extension Assay technology (OLINK) was used to further characterize the phenotype of Mphs and mDCs.

For secretome analysis using OLINK technology, ASC-conditioned media samples were sent to Firalis (Huningue, France) for analysis of their composition in the different conditions tested. The selected panel of OLINK targets for this study was the “inflammation panel” [29] (Olink Proteomics, Uppsala, Sweden), including 92 soluble, inflammation-related factors.

### Neutralization assays

To carry out neutralization experiments in vitro, mouse monoclonal anti-human IL-6 neutralizing antibody (clone MQ2-13A5 from eBioscience, Thermo Fisher Scientific, Waltham, MA, USA) or its corresponding isotype control immunoglobulin G1 were added to the co-culture of mDCs and ASCs at 10 μg/mL. Additionally, to block prostaglandin E2 (PGE2) secretion via cyclooxygenase (COX)-2 inhibition, we used indomethacin at 20 μM. The stock indomethacin (Millipore Sigma, St. Louis, MO, USA) was prepared in 100% ethanol, so equivalent volumes of ethanol were used in the co-cultures as carrier control. The co-cultures were spiked with anti-IL-6, indomethacin, or their controls at day 0 and day 3, and analyzed at day 7 for phagocytic and phenotypic studies.

### Generation of genetically modified ASCs for COX-2

COX-2 KO ASC lines were generated via CRISPR gene editing. Guided RNA was designed to target COX-2 exon 3 (CGTTCCAAAATCCCTTGAAG). For delivery, the Alt-R^®^ CRISPR-Cas9 System was used following the manufacturer’s instructions (Integrated DNA Technologies [IDT], IA, USA), and the protocol was optimized for the lipofection of 1.5 million cells per condition. Briefly, ASCs were seeded in T-75 flasks at a density of 30,000 cells/cm^2^ and allowed to rest overnight. Then, 1.5 million cells were lipofected and maintained 48 h with the lipofection complexes containing Lipofectamine™ RNAiMAX Transfection Reagent (Thermo Fisher Scientific, Waltham, MA, USA), Alt-R^®^ CRISPR-Cas9 crRNA XT (IDT, IA, USA), Alt-R^®^ CRISPR-Cas9 tracrRNA (IDT, IA, USA), and Alt-R^®^ S.p. HiFi Cas9 Nuclease V3 (IDT, IA, USA). Parental ASCs were transfected with lipofection complexes without tracrRNA (trans-activating CRISPR RNA) and crRNA (CRISPR RNA). Lipofection efficiency was evaluated at 48 h through the trans-activating crRNA labeled with ATTO-550 fluorophore using a Leica fluorescence microscope (Leica Microsystems, Wetzlar, Germany); cells were harvested, counted, and plated on transwell inserts for co-culture experiments.

To evaluate the efficiency of the COX-2 KO procedure, a portion of the lipofected cells were seeded at 10,000 cells/cm^2^ in T-25 flasks for 24 h. ASCs were then stimulated with 10 ng/mL IL-1β for 4 h or left untreated. ASC supernatants were collected and frozen at − 80 °C for ELISA analysis, and cells were harvested and frozen for quantitative polymerase chain reaction (qPCR) analysis.

RNA from cells was obtained using RNeasy Mini Kit (Qiagen, Hilden, Germany), and cDNA was synthetized from messenger RNA using SuperScript^®^ III Reverse Transcriptase (Thermo Fisher Scientific, Waltham, MA, USA). Finally, qPCR was performed with primers for *COX-2* (Fw, CGCAAACGCTTTATGCTGAA; Rv, CTCCACAGCATCGATGTCAC) and *GADPH* (Fw, CTCCTGTTCGACAGTCAGC; Rv, CCCAATACGACCAAATCCGT) (IDT, IA, USA), using a SYBR™ Green PCR Master Mix and a qPCR One Step Plus equipment (Thermo Fisher Scientific, Waltham, MA, USA).

PGE2 levels from culture supernatants were determined by ELISA using a PGE2 ELISA Kit-Monoclonal (Cayman Chemical, Ann Arbor, MI, USA) according to the manufacturer’s instructions. All determinations were made in triplicates. The absorbance was determined using an Envision Multilabel Reader (PerkinElmer, Waltham, MA, USA), and the results were analyzed using the GraphPad Software.

### Generation of ASC-conditioned media

ASCs were seeded in T-175 flasks at 18,000 cells/cm^2^ and incubated in complete Dulbecco’s modified Eagle’s medium (DMEMc). After 24 h of resting, the media were replaced for DMEMc alone or DMEMc with 10 ng/mL IL-1β and incubated for 6 h. Subsequently, the media were removed, cells were washed once with PBS, and 30 mL of fresh RPMIc was added to each culture condition to generate control and IL-1β-conditioned media. 48 h later, basal and IL-1β-conditioned media were collected and frozen at − 80 °C until its use. PGE2 levels were measured by an ELISA assay following the manufacturer’s instructions (Cayman Chemical, Ann Arbor, MI, USA).

For the conditioned medium assay, monocyte differentiation to mDC was performed as previously indicated, but co-culture with ASCs was replaced by the addition of control or IL-1β-conditioned media at days 0, 3, and 5 of the differentiation process. mDCs were analyzed at day 7 for phagocytic and phenotypic parameters.

### Statistical analysis

For phagocytosis and phenotypic analysis by flow cytometry, at least three independent experiments were performed. Average values of % positive or average values of the geometric mean of fluorescence for the specific markers after subtraction of the isotype control value are represented, plus standard deviation. Statistical significance between monocytes in non-contact conditions and monocytes in co-culture with ASCs was assessed using Student’s *t* test. The same test was used in conditioned medium experiments.

Statistical analysis of the secretome assessment was performed at Firalis using the software R version 3.3.3 and its associated packages, as well as FactoMineR version 1.36. Missing data below the limit of detection (LOD) was replaced by LOD value for further analysis. Before beginning the statistical analysis, the following descriptive statistics were performed on each continuous variable: minimum and maximum, percentiles (5%, 25%, 50%, 75%, 95%), mean, variance and standard deviation, and range and interquartile range. Boxplots were also used to visualize the distribution of each variable. To compare the group two by two, we applied a non-parametric Wilcoxon–Mann–Whitney test and compared the fold changes between the groups. Significance was typically determined by a *p* value below 0.05 from the Wilcoxon–Mann–Whitney test and a fold change between the groups either superior to 2 or inferior to 0.5. To determine the statistical significance in CRISPR/Cas9 assays, a 1-way ANOVA test was performed.

## Results

### ASCs induce morphological changes and increased survival in Mphs and mDCs

**Fig. 1:**
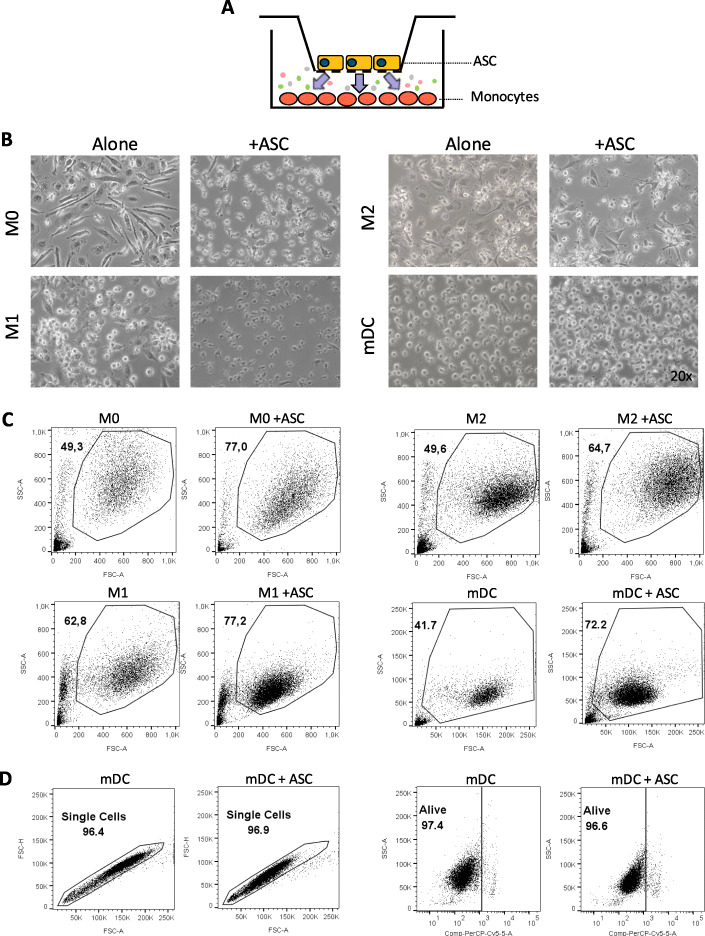
Morphology changes of monocyte-derived populations by ASCs. **a** Transwell setting of ASC and monocyte co-cultures. **b** Bright-field microscopy pictures of Mphs or mDCs in the presence or absence of ASCs at × 20 magnification. **c** FSC/SSC analysis by flow cytometry of M0, M1, and M2 Mphs, and mDCs in the absence or presence of ASCs. **d** FSC-Height versus FSC-Area to select singlets on mDC population (left) and 7-AAD staining to exclude dead cells (right). Data are representative of at least four independent experiments. Numbers in **c** represent the percentage of the gated population in the total sample. ASC, adipose-derived mesenchymal stem cell; FSC, forward scatter; mDC, mature dendritic cell; Mph, macrophage; SSC, side scatter

In order to characterize the ability of ASCs in vitro to modulate the differentiation and function of monocytes and their derivatives, we induced differentiation of isolated CD14^+^ CD16^−^ monocytes toward non-polarized M0 Mphs. Alternatively, we induced polarization toward M1 pro-inflammatory Mphs, M2 anti-inflammatory Mphs, or mDCs. These inductions were done in the presence or absence of ASCs, in non-contact conditions (Fig. 1a). In the presence of ASCs, a change in the morphology of all myeloid cells was observed, which was especially visible for non-polarized M0 and M1 pro-inflammatory Mphs, both under the microscope (Fig. 1b) and by flow cytometry (Fig. 1c).

In the presence of ASCs, cell viability was increased at the end of the differentiation process, and this was accompanied by a reduction in the abundance of cell debris. During flow cytometry analysis, when ASCs were present, there were higher proportions of Mphs and, particularly, mDCs falling into the live gating path (Fig. 1c). We also excluded doublets through the FSC-H/FSC-A (forward scatter height versus area) analysis, as shown in Fig. 1d. The percentage of 7-AAD-negative—and therefore viable—cells in the live selected gate was over 95% in all cases (Fig. 1d).

Next, the influence of ASCs on the phenotype, phagocytic capacity, and inflammatory secretome of the four different myeloid cell types was investigated.

### ASCs enhance the phagocytic capacity and anti-inflammatory profile of M0 non-polarized Mphs

**Fig. 2:**
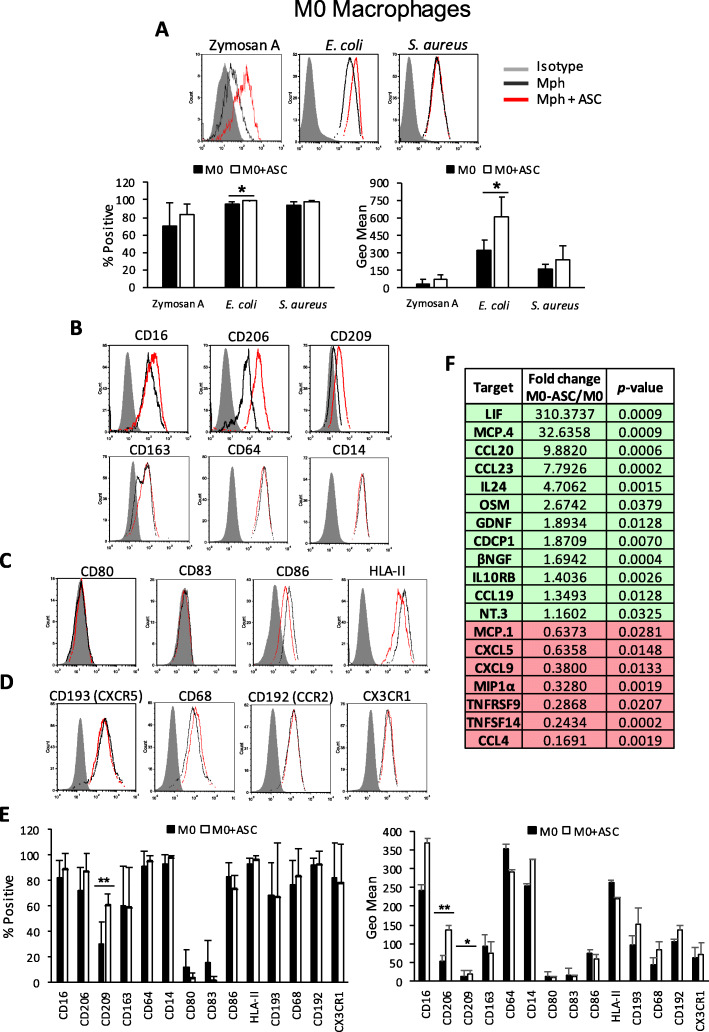
Phenotypic and functional analysis of ASC-educated M0 Mphs. Flow cytometry analysis of **a** phagocytosis levels of Zymosan A, *E. coli*, or *S. aureus* particles labeled with pHrodo™. Average ± SEM of positive percentages and Geo Mean statistics are shown below; **b** surface expression of several phagocytic receptors; **c** surface expression of co-stimulatory molecules; **d** surface chemokine receptors, in M0 Mphs, in the presence or absence of ASCs; and **e** Average ± SEM of positive percentages and Geo Mean of the different surface markers. **f** OLINK analysis of the secretome of M0 Mphs alone, and M0 Mph and ASC co-cultures. Data are representative of at least three independent experiments. The table shows the fold change of NPX between M0 Mphs with ASCs and M0 Mphs alone, together with the *p* value for this calculation; green indicates the upregulation of targets, and red indicates the downregulation of targets; only statistically significant changes are shown (*n* = 4). *p < 0.05, **p < 0.01. ASC, adipose-derived mesenchymal stem cell; CCL, C-C motif chemokine; CCR, C-C motif chemokine receptor; CD, cluster of differentiation; CDCP, CUB domain-containing protein; CX3CR, CX3C chemokine receptor; CXCL, C-C-C motif chemokine; CXCR, C-X-C chemokine receptor; *E. coli*, *Escherichia coli*; GDNF, glial cell line-derived neurotrophic factor; HLA, human leukocyte antigen; IL, interleukin; IL10RB, IL-10 receptor subunit beta; LIF, leukemia inhibitory factor; MCP, monocyte chemotactic protein; MIP, macrophage inflammatory protein; Mph, macrophage; NGF, nerve growth factor; NPX, normalized protein expression; NT-3, neurotrophin-3; OSM, oncostatin-M; *S. aureus*, *Staphylococcus aureus*; TNFRSF, tumor necrosis factor receptor superfamily member; TNFSF, tumor necrosis factor ligand superfamily member

M0 Mphs are non-polarized Mphs that arise from monocytes migrating to peripheral tissues and that have differentiated into Mphs in the presence of M-CSF or GM-CSF. However, they have not encountered specific factors in order to polarize them toward either a pro- or anti-inflammatory profile. To study the effect of ASCs on their phagocytic ability, we incubated M0 Mphs (both in the presence and absence of ASC) with Zymosan A, *E. coli*, and *S. aureus* pHrodo™ particles and analyzed by flow cytometry. In the presence of ASCs, the total percentage and/or the value of geometric mean fluorescence intensity of the positive peaks of phagocytic M0 Mphs for *E. coli* particles was increased (Fig. 2a) compared with M0 Mphs which were differentiated in the absence of ASCs. These data demonstrate that the co-culture of monocytes in the presence of ASCs results in M0 Mphs with an enhanced in vitro phagocytic potential toward bacterial particles.

To further characterize the enhanced phagocytic function of M0 Mphs in the presence of ASCs, the surface expression of Mph phagocytic receptors was analyzed by flow cytometry. The co-culture of monocytes in the presence of ASCs resulted in a moderate upregulation CD209 receptor, involved in phagocytosis of opsonized pathogens and apoptotic cells (Fig. 2b, e), on the cell surface of M0 Mphs. The co-culture of ASCs and monocytes resulted in a greater increase in CD206 surface expression, a receptor that mediates the internalization of a broad range of bacteria, fungi, and parasites (Fig. 2b, e). The expression of CD163 and CD64 was not altered in the presence of ASCs (Fig. 2b, e). The data support that the observed increase in the phagocytic capacity of M0 Mphs differentiated in the presence of ASCs likely relates to the upregulation of cell surface CD206 and CD209 receptors.

The cell surface expression of co-stimulatory molecules implicated in the activation of other immune cell populations, such as human leukocyte antigen (HLA)-II, CD80, CD83, and CD86, was analyzed using flow cytometry. The co-culture of M0 Mphs with ASCs caused a tendency to decrease the expression of CD86 and HLA-II, but it was not statistically significant due to the variability between buffy coats (Fig. 2c, e). The expression of several chemokine receptors involved in Mph and DC trafficking and migration was also analyzed, and ASC co-culture did not modify the already high expression of CD193 (C-X-C chemokine receptor [CXCR] 5), CD68, CD192 (C-C motif chemokine receptor [CCR] 2), and CX3C chemokine receptor (CX3CR) 1 on the surface of M0 Mphs (Fig. 2d, e). Finally, the modulatory capacity of ASCs on the secretome of differentiated Mphs or mDCs was explored by measuring the levels of a panel of 92 soluble inflammatory factors using the OLINK technology. Of the 92 factors measured, the secretome of M0 Mph contained 53, with IL-8, monocyte chemotactic protein (MCP) 1, IL-6, C-X-C motif chemokine [CXCL] 1, CXCL5, CXCL10, and CSF1 being the most predominant (Suppl. Fig. 4). The presence of ASCs in the co-culture significantly increased and reduced the levels of 12 and 7 factors, respectively (Fig. 2f). Leukemia inhibitory factor (LIF), MCP4, C-C motif chemokine (CCL) 20, CCL23, and IL-24 were highly increased by ASC co-culture, while CCL4, Mph inflammatory protein (MIP) 1a, MCP1, and some TNF-related molecules were highly reduced. Therefore, ASCs modulated the secretome of M0 Mph toward a more anti-inflammatory profile.

### ASCs modulate M1 Mphs toward a phagocytic and anti-inflammatory profile

**Fig. 3:**
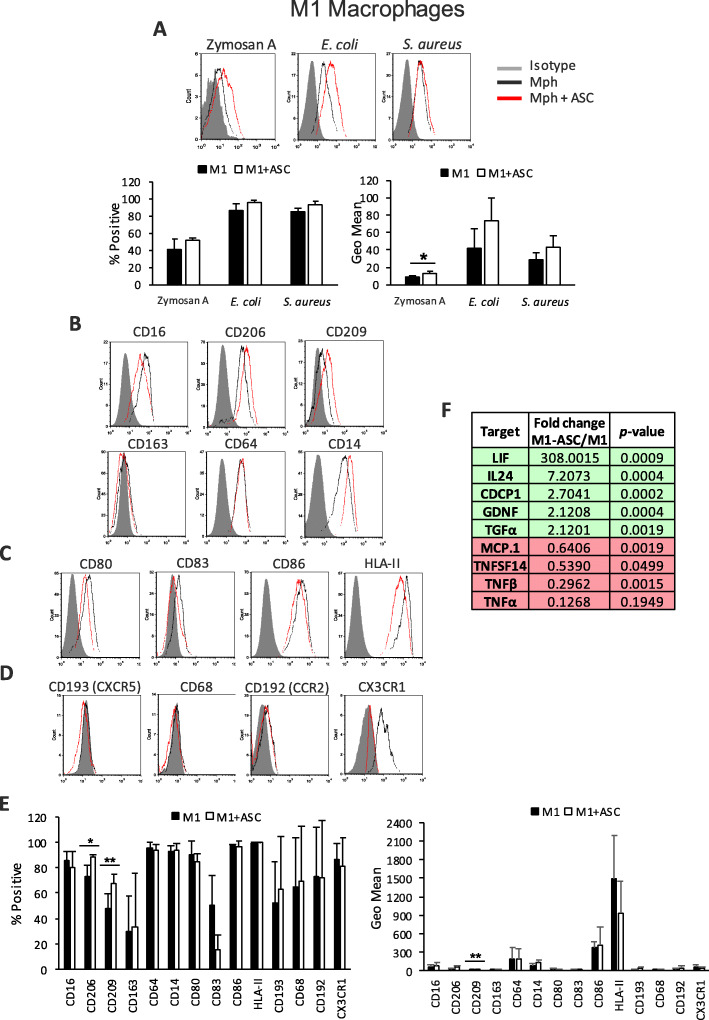
Phenotypic and functional analysis of ASC-educated M1 Mphs. Flow cytometry analysis of **a** phagocytosis levels of Zymosan A, *E. coli*, or *S. aureus* particles labeled with pHrodo™. Average ± SEM of positive percentages and Geo Mean statistics are shown below; **b** surface expression of several phagocytic receptors; **c** surface expression of co-stimulatory molecules; **d** surface chemokine receptors, in M1 Mphs, in the presence or absence of ASCs; and **e** Average ± SEM of positive percentages and Geo Mean of the different surface markers. **f** OLINK analysis of the secretome of M1 and ASC-M1 Mph co-cultures. Data are representative of at least three independent experiments. The table shows the fold change of NPX between M1 Mphs + ASCs and M1 alone, together with the *p* value for this calculation; green indicates the upregulation of targets, and red indicates the downregulation of targets; only statistically significant changes are shown (*n* = 4). *p < 0.05, **p < 0.01. ASC, adipose-derived mesenchymal stem cell; CCR, C-C motif chemokine receptor; CD, cluster of differentiation; CDCP, CUB domain-containing protein; CX3CR, CX3C chemokine receptor; CXCR, C-X-C chemokine receptor; *E. coli*, *Escherichia coli*; GDNF, glial cell line-derived neurotrophic factor; HLA, human leukocyte antigen; IL, interleukin; LIF, leukemia inhibitory factor; MCP, monocyte chemotactic protein; Mph, macrophage; NPX, normalized protein expression; *S. aureus*, *Staphylococcus aureus*; TGF, transforming growth factor; TNF, tumor necrosis factor; TNFSF, tumor necrosis factor ligand superfamily member

M1 Mphs are classically activated Mphs that are generated in peripheral tissues upon TLR and/or IFN signaling, which occurs during acute inflammatory conditions and infections. M1 Mphs have limited phagocytic capacity and elicit a pro-inflammatory profile, acting as the first line of defense against intracellular pathogens, promoting activation of the Th1 lymphocyte axis [30, 31]. As expected, M1 Mphs in the absence of ASCs showed a moderate capacity to phagocytose *E. coli*, *S. aureus*, and, to a lesser extent, Zymosan A particles (Fig. 3a). The presence of ASCs increased the phagocytic properties of M1 Mphs toward Zymosan A (Fig. 3a). The distinctive surface profile of M1 Mphs is CD64^+^ CD80^+^. In our phenotypic analysis, M1 Mphs also expressed CD14, CD16, CD206, and CD209 but, in contrast to M0 Mphs, did not express CD163 (Fig. 3b). ASCs had a similar effect on the expression of these receptors in M1 Mphs to what we observed in non-polarized M0 Mphs; the expression of CD206 and CD209 was consistently induced by ASCs. Similar to M0 Mphs, the expression of CD64 and CD163 was not affected by the ASC co-culture (Fig. 3b, e). The enhanced phagocytic capacity of M1 Mphs co-cultured with ASCs toward Zymosan A is likely due to the increased expression of CD206 and CD209 cell surface receptors.

M1 Mphs typically express not only CD86 and HLA-II, as M0 Mphs do, but also CD80 and CD83 (Fig. 3c, e). The co-culture of M1 Mphs with ASCs did not cause statistically significant downregulation of any of these molecules (Fig. 3c, e). Additionally, the basal chemokine receptor expression profile of M1 Mphs was distinct to non-polarized M0 Mphs, with M1 Mphs only expressing CX3CR1 (fractalkine receptor) and CCR2, to a lesser extent. The presence of ASCs did not cause any significant changes (Fig. 3d, e). When analyzing the secretome of M1 Mphs, 58 of the panel of 92 factors measured were detected, with TNFα, IL-6, IL-8, CCL4, CD40, CSF1, CXCL1, CXCL10, CXCL11, and MCP1–4 being among the most secreted (Suppl. Fig. 4). In the presence of ASCs, nine secreted proteins were significantly modulated. We observed an increase of LIF, IL-24, CUB (for complement C1r/C1s, Uegf, Bmp1) domain-containing protein (CDCP) 1, glial cell line-derived neurotrophic factor (GDNF), and transforming growth factor (TGF)-α, while inflammatory factors such as TNFα, TNFβ, MCP1, and TNF ligand superfamily member (SF) 14 were highly decreased (Fig. 3f). These data indicate that in the presence of ASCs, M1 pro-inflammatory Mphs were modulated toward a more phagocytic, less stimulatory profile, with a marked anti-inflammatory secretome.

After establishing the ability of ASCs to modulate M1 Mphs along their polarization process, we investigated their capacity to revert polarization of M1 Mphs once fully differentiated, a situation that ASCs would encounter when targeting already inflamed tissues in vivo. Firstly, monocytes were fully differentiated toward M1 Mphs (in the absence of ASC), and ASCs were then incorporated in a transwell co-culture for 4 additional days (Suppl. Fig. 1A). The results demonstrated that, in our in vitro experimental settings, ASCs were not able to revert the pro-inflammatory phenotype of fully polarized M1 Mphs nor modify their phagocytic profile (Suppl. Fig. 1B and C).

### ASCs enhance the phagocytic capacity and anti-inflammatory profile of M2 Mphs

Cytokines such as IL-4, IL-13, and IL-10, induced during a Th2 response, modulate non-polarized M0 Mphs toward the anti-inflammatory profile of M2 Mphs [32]. There are at least three subtypes of M2 Mphs: M2a-like, M2b-like, and M2c-like [32]. Each subtype has slightly different cell surface proteins and secretomes, but in general, they exhibit anti-inflammatory and reparative profiles [32]. In our experimental settings, we obtain an M2a-like population, characterized by a lack of CD14 and the expression of CD206, CD209, CD163, and CD86 cell surface receptors.

**Fig. 4:**
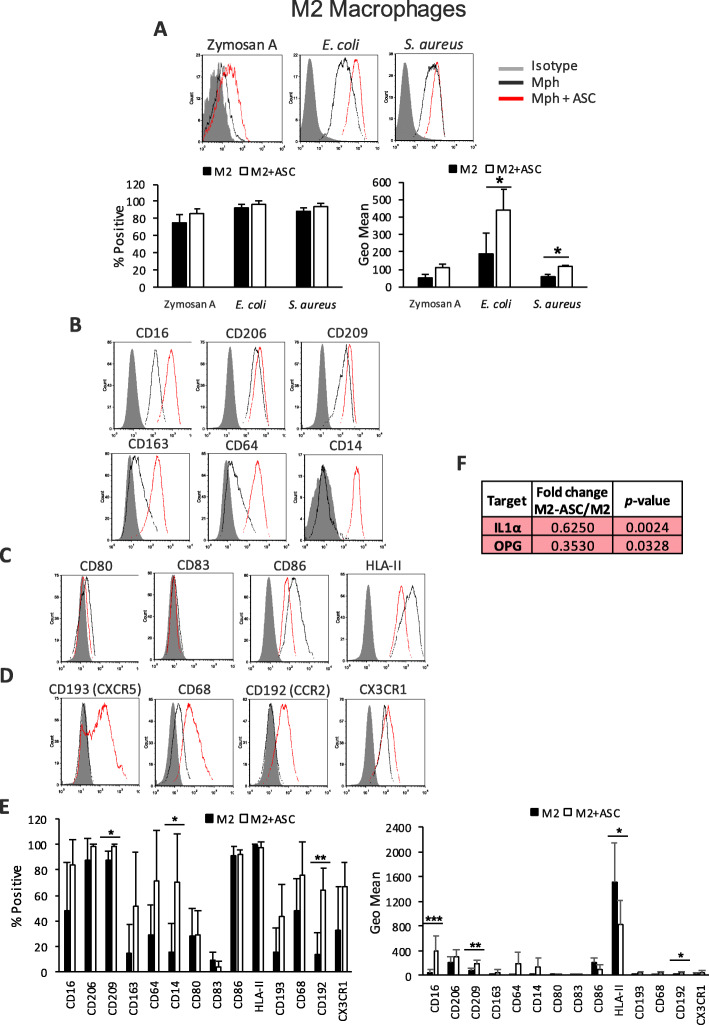
Phenotypic and functional analysis of ASC-educated M2 Mphs. Flow cytometry analysis of **a** phagocytosis levels of Zymosan A, *E. coli*, or *S. aureus* particles labeled with pHrodo™. Average ± SEM of positive percentages and Geo Mean statistics are shown below; **b** surface expression of several phagocytic receptors; **c** surface expression of co-stimulatory molecules; **d** surface chemokine receptors, in M2 Mphs, in the presence or absence of ASCs; and **e** Average ± SEM of positive percentages and Geo Mean of the different surface markers. **f** OLINK analysis of the secretome of M2 Mphs alone and ASC-M2 Mphs co-cultures. Data are representative of at least four independent experiments. The table shows the fold change of NPX between ASC-M2 Mphs and M1 alone, together with the *p* value for this calculation; red indicates the downregulation of targets; only statistically significant changes are shown (*n* = 4). *p < 0.05. ASC, adipose-derived mesenchymal stem cell; CCR, C-C motif chemokine receptor; CD, cluster of differentiation; CX3CR, CX3C chemokine receptor; CXCR, C-X-C chemokine receptor; *E. coli*, *Escherichia coli*; HLA, human leukocyte antigen; IL, interleukin; Mph, macrophage; NPX, normalized protein expression; OPG, osteoprotegerin; *S. aureus*, *Staphylococcus aureus*

As expected, when co-cultured with ASCs, M2 Mphs demonstrated an enhanced phagocytic capacity toward *E. coli* and *S. aureus* particles (Fig. 4a). Like M0 Mphs, M2 Mphs express CD16, CD206, CD209, CD163, and CD64, with CD209 showing higher expression levels, while CD163 and CD64 showed lower levels compared with M0 Mphs. In contrast to M0 Mphs, M2 Mphs did not express CD14. In the presence of ASCs, we observed an increase of CD16 and CD209 on the surface of differentiated M2 Mphs, compared with control. As mentioned, M2 Mphs are characterized by a lack of CD14; however, CD14 was strongly induced de novo in M2 Mphs co-cultured with ASCs (Fig. 4b, e). In addition to this, the expression of HLA-II on the surface of M2 Mphs was downregulated in the presence of ASCs (Fig. 4c, e). The chemokine receptor profile of M2 Mphs shared some characteristics with M0 Mphs, for example, expression of CD68 and CX3CR1—chemokine receptors associated with reparative functions. They also shared some characteristics with M1 Mphs, such as lack of CXCR5 and CCR2 expression (Fig. 4d, e). ASC-modulated M2 Mphs expressed de novo CCR2 which may have an impact on the migratory profile of these cells in vivo toward MCP1–4 (Fig. 4d, e). The secretome of M2 Mph contained 58 of the 92 soluble factors screened, with IL-8, P1, IL-6, CXCL1, CXCL5, CCL23, CCL4, osteoprotegerin (OPG), TGF-α, and CSF1 being among the most produced (Suppl. Fig. 4). When co-cultured with ASCs, only IL-1α and OPG were significantly reduced (Fig. 4f).

### ASCs mediate the generation of a new regulatory and highly phagocytic mDC population

**Fig. 5:**
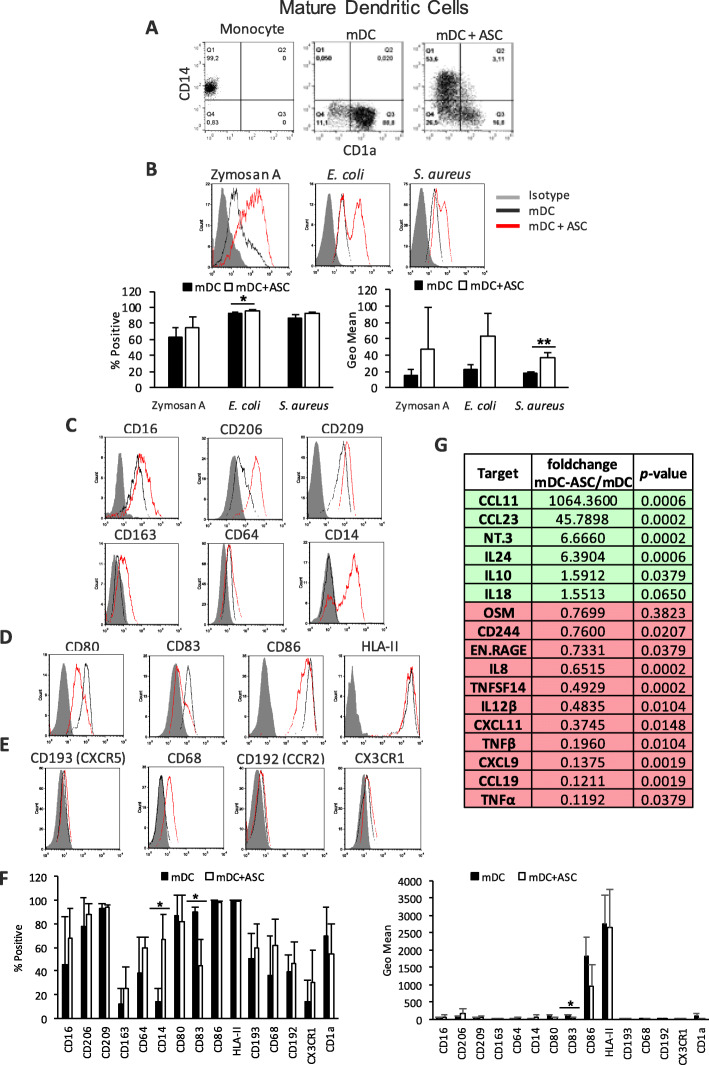
Phenotypic and functional analysis of ASC-educated mDCs. Flow cytometry analysis of **a** surface expression of CD14/CD1a; **b** phagocytosis levels of Zymosan A, *E. coli*, or *S. aureus* particles labeled with pHrodo™. Average ± SEM of positive percentages and Geo Mean statistics are shown below; **c** surface expression of several phagocytic receptors; **d** surface expression of co-stimulatory molecules; and **e** surface chemokine receptors in mDCs in the presence or absence of ASCs. **f** Average ± SEM of positive percentages and Geo Mean of the different surface markers. Data are representative of at least four independent experiments; **g** OLINK analysis of the secretome of mDCs alone and ASC–mDC co-cultures. The table shows fold change of NPX between ASC–mDC and mDCs alone, together with the *p* value for this calculation; green indicates the upregulation of targets, and red indicates the downregulation of targets; only statistically significant changes are shown (*n* = 4). *p < 0.05, **p < 0.01. ASC, adipose-derived mesenchymal stem cell; CCL, C-C motif chemokine; CCR, C-C motif chemokine receptor; CD, cluster of differentiation; CX3CR, CX3C chemokine receptor; CXCL, C-C-C motif chemokine; CXCR, C-X-C chemokine receptor; *E. coli*, *Escherichia coli*; EN.RAGE, protein S100-A12; HLA, human leukocyte antigen; IL, interleukin; mDC, mature dendritic cell; NPX, normalized protein expression; NT-3, neurotrophin-3; OSM, oncostatin-M; *S. aureus*, *Staphylococcus aureus*; TNF, tumor necrosis factor; TNFSF, tumor necrosis factor ligand superfamily member

DCs are antigen-presenting cells that are able to capture self and pathogenic antigens, process them, and present them, conjugated with major histocompatibility complexes, to T cells [33, 34]. They also regulate the function of T-lymphocyte populations and are capable of activating regulatory T cells and inducing apoptosis of autoreactive T cells [33, 34]. Different DC subsets with different profiles exert specific pro-inflammatory or regulatory functions. During the differentiation process from monocytes, iDCs downregulate CD14 and begin expressing CD1a, a surface molecule that is not expressed in any other monocyte-derived population and is implicated in the antigen presentation process [34]. Typically, fully differentiated DCs are CD14^−^ CD1a^+^ (Fig. 5a) [33, 35]. Yet, in the presence of ASCs, a novel CD14^+^ CD1a^−^ population was generated (Fig. 5a). This newly formed mDC population has previously been attributed to higher anti-inflammatory properties and regulatory functions compared with the typical mDC population [35]. The modulatory effect of ASCs was partial, with two distinct mDC populations being detected.

The phagocytic capacity of mDCs co-cultured with ASCs was considerably enhanced through the generation of a new, highly phagocytic mDC population (Fig. 5b). The presence of ASCs during differentiation caused the appearance of a new mDC population, highly phagocytic toward bacterial particles from both for *E. coli* and *S. aureus* (Fig. 5b). mDCs express CD16, CD206, CD209, and, to a lesser extent, CD64 on their cell surface, but they lack CD163 and CD14. The co-culture of mDCs with ASCs induced the expression of CD14 within a subset of cells (Fig. 5c).

In the absence of ASCs, mDCs highly expressed all the co-stimulatory molecules analyzed in this study, and the effect of ASC co-culture caused a downregulation of CD83 (Fig. 5d, f). Additionally, mDCs showed a different chemokine receptor profile compared with M0 Mphs and lacked expression of CXCR5, CD68, and CCR2, with minimal expression of CX3CR1. When in the presence of ASCs, the expression of the chemokine receptors analyzed was not significantly modified (Fig. 5e, f). The secretome of mDCs contained 59 of the 92 soluble factors screened, with CCL4, CD40, CXCL11, CXCL5, CXCL9, IL-6, IL-8, IL-12β, MCP1, MCP2, MIP1α, and TNFα being among the most produced (Suppl. Fig. 4). In the presence of ASCs, the secretion of six soluble factors—CCL11, CCL23, neurotrophin-3, IL-24, IL-18, and IL-10—was increased, and 11 factors, including TNFα, TNFβ, IL-8, TNFSF14 (tumor necrosis factor ligand superfamily member 14), CCL19, and CXCL9, were reduced (Fig. 5g). These data suggest that co-culture with ASCs modulated the mDC secretome toward a more anti-inflammatory profile.

### Neutralization of PGE2 abolished the modulatory effect of ASCs on mDC phenotype and function

**Fig. 6:**
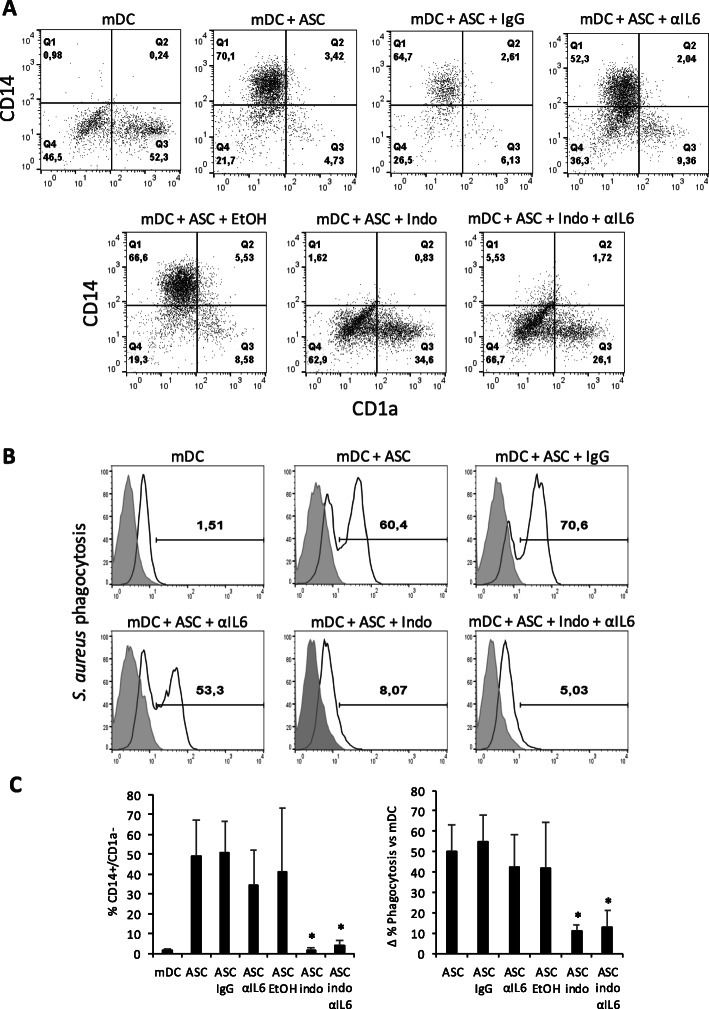
CD14/CD1a surface expression and phagocytic capacity of ASC-educated mDCs are modulated by IL-6 and PGE2. **a** Dot plots showing the surface expression of CD14/CD1a, in monocyte-derived mDCs in the presence or absence of ASCs and modulated by IL-6 or PGE2 inhibitors, measured by flow cytometry (*n* = 3). **b** Histograms show the phagocytosis levels of *S. aureus* particles by monocyte-derived mDCs, in the presence or absence of ASCs, and modulated by IL-6 or PGE2 inhibitors, measured by flow cytometry. **c** Graphs showing the average percentage of CD14^+^/CD1a^−^ ± SEM (left) and the average increase of the percentage of phagocytic cells ± SEM (right) (*n* = 3). IgG1 was the negative control for αIL-6, and ethanol was added as an indomethacin carrier. *p < 0.05. ASC, adipose-derived mesenchymal stem cell; CD, cluster of differentiation; EtOH, ethanol; IgG, immunoglobulin G; Indo, indomethacin; IL, interleukin; mDC, mature dendritic cell; PGE2, prostaglandin E2; *S. aureus*, *Staphylococcus aureus*

Previous studies have reported that both PGE2 and IL-6 may play a role in MSC-mediated Mph and DC modulation [36]. We carried out experiments in which monocytes were differentiated into mDCs with or without ASCs, in the presence or absence of a neutralizing antibody against IL-6 and/or indomethacin, a COX-1/2 chemical inhibitor that blocks PGE2 synthesis. The phenotype and phagocytic properties of mDCs were then analyzed. Neutralization of PGE2 with indomethacin completely inhibited the ability of ASCs to modulate the differentiation of monocytes toward mDCs (Fig. 6a, c). However, anti-IL-6 resulted in only a minor reduction in the modulatory effect of ASCs on the phagocytic properties of mDCs toward *S. aureus* particles that were not statistically significant (Fig. 6b, c). Neutralization of IL-6 partially reduced the induction of CD206, but the addition of indomethacin completely inhibited its induction and reverted its expression to basal levels of mDCs (Suppl. Fig. 2A). When investigating this effect on CD209 expression, neither indomethacin nor anti-IL-6 alone inhibited ASC-related CD209 upregulation completely, but their combination restored CD209 to basal levels (Suppl. Fig. 2B). The increase in CD163 expression induced by ASCs was partially blocked with anti-IL-6 and completely abrogated with indomethacin (Suppl. Fig. 2C); this was also the case with co-stimulatory molecules CD80, CD86, and HLA-II (not shown). These results demonstrate that PGE2 and, to a certain extent, IL-6 are able to mediate the modulatory effect of ASCs on the phenotype and phagocytic function of mDCs during in vitro differentiation.

### Knock-down of COX-2 in ASCs confirms that PGE2 mediating myeloid modulation is secreted by ASCs

**Fig. 7:**
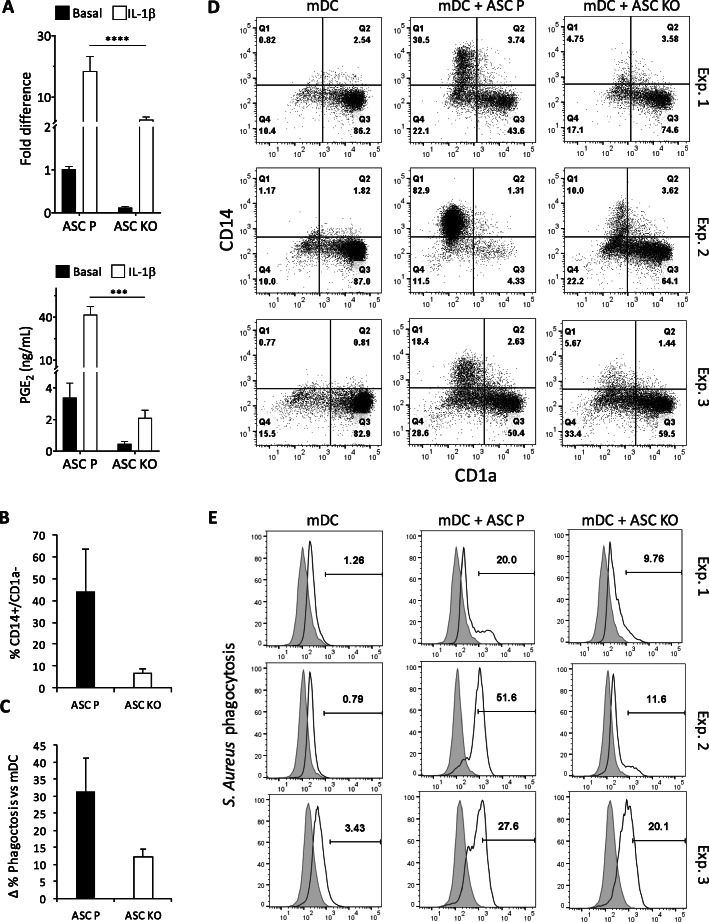
CD14/CD1a surface expression and phagocytic capacity of mDCs co-cultured with COX-2 KO or parental ASCs. **a** COX-2 qPCR (top) and PGE2 ELISA (bottom) of COX-2 KO or parental ASCs in basal conditions or upon IL-1β stimulation (*n = 3*). **b**, **d** Dot plots and graphs showing the surface expression of CD14/CD1a, in monocyte-derived mDCs in the presence or absence of COX-2 KO or parental ASCs, measured by flow cytometry (*n* = 3). Average positive percentage ± SEM is represented. **c**, **e** Histograms and graphs showing the phagocytosis levels of *S. aureus* by monocyte-derived mDCs, in the presence or absence of COX-2 KO or parental ASCs, measured by flow cytometry (*n* = 3). Average positive percentage increase versus mDC alone ± SEM is represented. ***p < 0.001, ****p < 0.0001. ASC, adipose-derived mesenchymal stem cell; CD, cluster of differentiation; COX-2, cyclooxygenase 2; Exp, experiment; KO, knock-out; mDC, mature dendritic cell; P, parental; PGE2, prostaglandin E2; *S. aureus*, *Staphylococcus aureus*

Once the key role of PGE2 in ASC-mediated myeloid modulation was demonstrated, we designed experiments to elucidate which cell population, monocytes/mDC or ASCs, was the secretor of PGE2. We targeted exon 3 of the COX-2 gene in ASCs using the CRISPR/Cas9 technology to knock down its expression and reduce or eliminate PGE2 secretion. First, to test the level of downregulation of COX-2, we stimulated parental ASCs and KO ASCs with IL-1β or left untreated and analyzed COX-2 expression levels by qPCR. IL-1β treatment mimics a pro-inflammatory environment and is a potent inductor of COX-2. We observed a minor decrease in COX-2 levels in unstimulated KO ASCs versus parental ASCs (Fig. 7a). However, this downregulation was more apparent when both cell types were stimulated with IL-1β; in this case, we found a significant 10-fold downregulation of COX-2 in KO ASCs versus parental ASCs (Fig. 7a). Subsequently, we analyzed the decrease in secreted PGE2 caused by COX-2 KO by ELISA. PGE2 levels were very low when ASCs were left untreated, observing a modest decrease upon COX-2 KO (Fig. 7a). We were able to significantly decrease PGE2 secretion by 20-fold after COX-2 KO when ASCs were stimulated with IL-1β (Fig. 7a).

We performed the differentiation of monocytes to mDC in the presence of KO ASCs, parental ASCs, or alone. Parental ASCs were able to induce CD14^+^/CD1a^−^ population. This induction was abrogated by the knock-down of COX-2, as shown in the co-cultures with KO ASCs (Fig. 7b, d). The phagocytosis of *S. aureus* particles was notably induced by parental ASCs, whereas this effect was considerably reduced when KO ASCs were added to mDC cultures (Fig. 7c, e). We also analyzed the effect of COX-2 KO in the expression of phagocytic markers on the surface of mDC. While CD206 and CD209 were not altered due to COX-2 KO, we observed a very clear effect on CD163. Parental ASCs induced CD163, while this effect was abrogated when KO ASCs were used (Suppl. Fig. 3A). We found a strong positive correlation between the upregulation of CD163 and the increase in the phagocytic potential of mDC (Suppl. Fig. 3B).

There is a noticeable variability between the three experimental repeats due to the use of different buffy coats and also the differences in the knock-down level of COX-2 between different runs. However, our experiments consistently demonstrated that reduction of COX-2 in ASCs, and, therefore, of PGE2 secretion, highly impacted the modulatory effect of ASCs. This data correlates with the results from the indomethacin experiments and further confirms the mechanism of ASC-mediated mDC modulation through PGE2 secretion.

### ASC-conditioned media recapitulates the effect of ASC co-culture

**Fig. 8:**
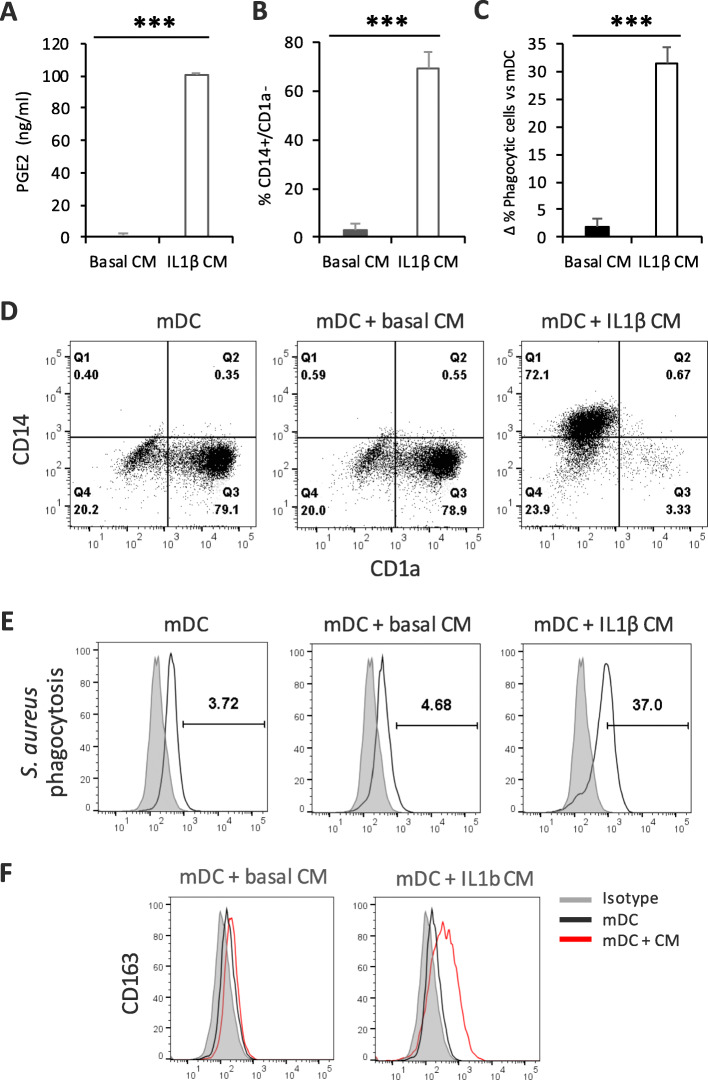
CD14/CD1a surface expression, phagocytic capacity, and CD163 expression of mDCs differentiated with ASC-conditioned supernatant. **a** PGE2 ELISA of basal or IL-1β-stimulated ASC-conditioned media. **b** Average positive percentage ± SEM of CD14^+^/CD1a^−^ mDC differentiated with basal or IL-1β-conditioned media (*n* = 3). **c** Average positive percentage increase of *S. aureus* phagocytosis versus mDC alone ± SEM of mDC differentiated with basal or IL-1β-conditioned media (*n* = 3). **d** Dot plots showing the surface expression of CD14/CD1a, in monocyte-derived mDCs in the presence of basal or IL-1β-conditioned media, measured by flow cytometry (data are representative of 3 independent experiments). **e** Histograms showing the phagocytosis levels of *S. aureus* by monocyte-derived mDCs, in the presence of basal or IL-1β-conditioned media, measured by flow cytometry (data are representative of 3 independent experiments). **f** Surface expression levels of CD163 on mDC differentiated in the presence of basal or IL-1β-conditioned media, measured by flow cytometry (data are representative of 3 independent experiments). ***p < 0.001. ASC, adipose-derived mesenchymal stem cell; CD, cluster of differentiation; CM, conditioned media; IL, interleukin; mDC, mature dendritic cell; PGE2, prostaglandin E2; *S. aureus*, *Staphylococcus aureus*

In order to further confirm the implication of soluble mediators secreted by ASCs in mDC modulation, we generated conditioned media from ASCs after IL-1β activation or in basal conditions. IL-1β is a pro-inflammatory cytokine secreted by monocytes and macrophages and is a potent inductor of PGE2. The PGE2 levels of these conditioned media were measured by ELISA. After IL-1β activation, PGE2 levels were about 100 ng/mL, but they were negligible in basal supernatant (Fig. 8a). We differentiated monocytes toward mDC in the presence of basal or IL-1β-conditioned media from ASCs. Basal-conditioned media were unable to induce CD14^+^/CD1a^−^ population while IL-1β-conditioned media had a very potent effect, inducing about 70% CD14^+^/CD1a^−^ population (Fig. 8b, d). Similarly, IL-1β-conditioned media induced high levels of *S. aureus* phagocytosis, while basal conditioned media had no effect (Fig. 8c, e). This increase in the phagocytic capacity of mDC toward bacterial particles correlated with a notable upregulation of CD163 (Fig. 8f).

These results confirm the key role of PGE2 in ASC-mediated myeloid modulation and corroborate that the secretion of PGE2 is originated by ASCs.

## Discussion

Evidence that the therapeutic effects of MSCs are mediated at least in part by the modulation of immune host cells, in particular monocyte and Mphs, is increasing [19–21, 24]. The mechanism by which MSCs modulate the differentiation and function of monocytes has been investigated mostly in MSCs from bone marrow and umbilical cord origin [13–15, 19, 24]. However, the current data with regard to the interaction and modulation of myeloid cells by ASCs are very limited. This study provides a greater understanding of the interactions between these cell types and contributes to our understanding of the potential therapeutic mechanisms of action.

Here, we evaluated the effect of ASCs on the in vitro differentiation and function of Mphs (M0, M1, and M2) and mDCs by co-culture in non-contact conditions during the differentiation process. We demonstrated that co-culture in the presence of ASCs increased the expression of phagocytic receptors in Mphs and mDCs, resulting in an enhanced phagocytic capacity. We also observed that the presence of ASCs resulted in the modulation of some surface chemokine receptors involved in the trafficking and migration of Mphs and mDCs. Finally, co-culture with ASCs enhanced the anti-inflammatory and antimicrobial capacity of the secretome of Mphs and mDCs compared with those in monoculture, with a decrease in the expression of co-stimulatory and pro-inflammatory secreted factors and an increase in anti-inflammatory factors.

Among all the populations analyzed, ASCs exerted the greatest modulatory effect in M0 Mphs and in mDCs, the former potentially demonstrating superior plasticity due to their neutral nature in terms of the inflammatory profile. The enhanced phagocytic capacity observed in all Mph populations and mDCs toward Zymosan A, *E. coli*, and *S. aureus* positively correlated with an increased expression of phagocytic receptors. CD206 (mannose receptor) and CD209 (DC-SIGN) are C-type lectins whose functions include phagocytosis of various bacteria, fungi, and parasites. CD163 or the haptoglobin–hemoglobin scavenger receptor is also implicated in phagocytosis and killing of several types of bacteria. It is also linked to wound healing and clearing of apoptotic cells in cases of tissue damage [37]. Another phagocytic receptor that was upregulated is CD14. CD14, the endotoxin receptor, is critical for TLR2-mediated M1 Mph activation. The main role of CD14 is to induce bacterial phagocytosis, although it is also involved in the clearance of apoptotic cells. Additionally, CD14 has been attributed to a role in the induction of survival of cells that express it [38]. Induction of its expression in anti-inflammatory M2 Mphs and mDCs co-cultured with ASCs may imply an ASC-mediated increased cell survival and resistance to cell death. ASCs modulated the expression levels on the surface of CD14 and CD1a in mDC. CD1a is tightly linked to the antigen-presenting function of DC. Presumably, these “ASC-educated” CD14^+^CD1a^−^ mDCs have been modified from activated antigen-presenting cells to phagocytic and regulatory cells [34].

Additionally, ASCs were able to induce some chemokine receptors in Mphs, such as CD192/CCR2. The upregulation of CD192/CCR2 may translate into an increased migratory potential of ASC-educated Mphs toward inflamed tissue, where levels of MCP4/CCL13 are elevated, to contribute to inflammation resolution.

ASCs were able to cause a deep transformation in the secretome of several Mph populations and mDC. The effects of ASCs on the secretome of M2 anti-inflammatory Mphs were very limited: only IL-1α and OPG were differentially secreted. The more moderate effect may be due to the innate anti-inflammatory nature of M2 Mphs. Some of the main factors upregulated in M0 and M1 Mph were LIF and IL-24. LIF is a cytokine that mediates differentiation and growth of different cell types (hematopoietic cells, neurons) but also acts as a chemoattractant for neutrophils, eosinophils, and monocytes, and it can induce IL-8 secretion; its production is induced by IL-6 [39–41], which is highly secreted by ASCs. IL-24 is a pleiotropic immunoregulatory cytokine, a member of the IL-10 family of cytokines. A role in the latest stages of wound healing has been attributed to IL-24 [42–44]. Also, MCP4 was induced in co-cultures of ASCs and M0 Mphs, compared to M0 alone. MCP4 is a chemoattractant for monocytes, eosinophils, T cells, and basophils [45, 46]. Some of the pro-inflammatory factors that are downregulated upon ASC co-culture are MIP1a, MCP1, MIP1b (CCL4), TNFα, and TNFβ, in M0 and M1 cultures. These results suggest that ASCs may potentially tilt the balance of pro- versus anti-inflammatory factors typical of M1 Mph secretome, which may have functional implications by limiting any ongoing exacerbated inflammation in infectious and inflammatory diseases. In the case of mDC, an increase in the IL-10/TNFα ratio indicates the anti-inflammatory nature of the regulatory mDC population induced by ASC modulation. The downregulation of TNFα, TNFβ, other TNF-related molecules, as well as other typically pro-inflammatory factors such as extracellular newly identified *receptor* for advanced glycation end-products binding protein (EN.RAGE), IL-8, and IL-12 clearly show how ASCs push mDC secretome toward a more anti-inflammatory balance. The presence of ASCs also downregulated the anti-inflammatory chemokines CCL23, CXCL9, and CXCL11. CCL23 is involved in mediating the chemotaxis of resting T cells and monocytes, whereas CXCL9 and CXCL11 both act as T cell chemoattractants. Overall, these in vitro data suggest that the infiltration of monocytes and activated lymphocytes to the site of inflammation could potentially be diminished with ASC-based therapies, as reported in experimental animal models in vivo [47–49].

Apart from the improvement in phagocytic function, secretome, and migratory properties, we also found a decrease in the co-stimulatory molecules CD83 and HLA-II in ASC-modulated macrophages and mDC. This may translate into a decreased ability of these populations to induce the activation of other immune cells.

Our data may, however, have some limitations. Firstly, it was performed in transwell conditions and without testing contact conditions. Therefore, it is not possible to know whether these conditions would have demonstrated the same modulatory effects with the same mechanisms implicated in contact conditions. Secondly, the secretome was analyzed during co-culture conditions with both ASCs and myeloid cells present, so it is not possible to specifically attribute a particular set of secreted factors to either of the two cell types individually. Lastly, due to the limited number of donors from which the ASCs and PBMCs were derived, it may not be possible to generalize these results, and further research is required.

From our neutralization studies, we can conclude that the different modulatory effects of ASCs on mDCs are mediated by PGE2 and, to a lesser extent, IL-6. A regulatory feedback loop has previously been reported, where PGE2 inhibition by indomethacin resulted in partial inhibition of IL-6 secretion from MSCs [50]. From the partial effect and lack of synergy observed in these experiments, it is feasible that a similar feedback loop may be operating here. In order to elucidate the origin of PGE2COX-2, knock-down experiments and conditioned medium experiments were performed, confirming the key role of PGE2 secreted by ASCs in their capacity to mediate myeloid modulation.

We unsuccessfully attempted to revert the pro-inflammatory properties of fully committed M1 Mphs, something that has not been previously published using MSC, to our knowledge. At least in our experimental settings, ASCs were able to modulate the differentiation of Mphs toward M1 phenotype, though were unable to modulate M1 Mphs once fully differentiated. We do not have a clear explanation for these results, but it might be due to the in vitro experimental conditions used that may need to be optimized (for instance, it may require cell-to-cell interactions, which were not tested here). Alternatively, it might be that the mechanisms by which ASCs modulate Mph function in vivo target the differentiation and polarization processes of infiltrating monocytes in the site of inflammation, but cannot modulate M1 Mphs once fully polarized. Further investigation is needed to clarify this relevant process.

In a therapeutic setting, our results imply that ASCs are able to generate myeloid cells with a reduced ability to activate local immune populations, as well as an altered migration pattern toward inflamed tissues, and an enhanced immunomodulatory and phagocytic capacity. The ASC-mediated increase of CD206, CD209, and CD163 on the surface of Mphs and mDCs suggests an enhanced ability to clear a range of blood-borne pathogens, supporting a therapeutic potential in infection-mediated diseases such as sepsis [51]. ASC-modulated Mphs may be able to reduce the microbial load in colitis and inflammatory bowel disease pathology [52]. Altogether, ASC-based therapy has the potential to provide a cell-mediated anti-inflammatory effect, with a decreased bacterial load and enhanced tissue repair and regeneration, such as fistula closure.

Currently, the use of ASC-based therapies provides a promising, novel approach to inflammatory conditions, and their potential continues to be explored. The data presented here provide more information on the mechanism of action of ASC therapies, such as darvadstrocel (https://www.ema.europa.eu/en/medicines/human/EPAR/alofisel), and help address gaps in our knowledge; this could facilitate studies on a wider application in clinical practice for a range of diseases, including different types of fistulas. The efficacy of ASCs is currently being explored for the treatment of severe, community-acquired bacterial pneumonia (NCT03158727), and the findings of the present study will provide valuable insights into this type of clinical trial. Further studies are necessary in order to fully characterize the mechanism of action of ASCs and accurately assess the efficacy and safety of ASCs in vivo.

## Conclusion

The data presented here demonstrate that ASCs are able to modulate the inflammatory phenotype and function of several myeloid populations during in vitro polarization. Mphs and mDCs differentiated in the presence of ASCs demonstrated an enhanced phagocytic and anti-inflammatory phenotype, characterized by an increase in phagocytic receptors on the cell surface, modulation of surface chemokine receptors toward an anti-inflammatory profile, and reduced expression of co-stimulatory molecules. Additionally, ASC co-culture promoted an anti-inflammatory, reparative, and antimicrobial phenotype of secreted proteins. The data demonstrate that mainly PGE2 and, to a lesser extent, IL-6 are the soluble factors responsible for this modulatory effect.

## Supplementary information

**Additional file 1: Suppl. Figure 1.** Reversion of M1 pro-inflammatory macrophage phenotype by ASC co-culture. A: Diagram shows the experimental settings of M1 Mph reversion assay using ASCs; B: Graphs show CD80/CD64 and HLA-II/CD86 surface expression of M1 Mph in the presence or absence of ASCs; C: Histograms showing the phagocytosis of *S. aureus* pHrodo™ particles and CD163, CD206 and CD209 expression by M1 Mphs in the presence or absence of ASCs, measured by flow cytometry. Data representative of two independent experiments. ASC, adipose-derived mesenchymal stem cell; CD, cluster of differentiation; IFN, interferon; LPS, lipopolysaccharide; M-CSF, Macrophage Colony-Stimulating Factor; Mph, macrophage; *S. aureus*, *Staphylococcus aureus*.

**Additional file 2: Suppl. Figure 2.** Surface expression levels of phagocytic receptors in ASC-educated mDCs modulated by IL-6 and PGE2 inhibitors. Histograms show the surface expression of A: CD206; B: CD209; and C: CD163, in monocyte-derived mDCs in the presence or absence of ASCs and modulated by IL-6 or PGE2 inhibitors, measured by flow cytometry. IgG1 was the negative control for αIL-6, and ethanol was added as indomethacin carrier (*n* = 3). ASC, adipose-derived mesenchymal stem cell; CD, cluster of differentiation; EtOH, ethanol; IgG, immunoglobulin G; Indo, indomethacin; IL, interleukin; mDC, mature dendritic cell; PGE2, prostaglandin E2.

**Additional file 3: Suppl. Figure 3.** CD163 surface expression of mDC differentiated in the presence of COX-2 KO ASCs or parental ASCs. A: Histograms show the surface expression of CD163 of mDC differentiated in the presence of COX-2 KO ASCs or parental ASCs; B: Correlation between CD163 expression and increase in percentage of *S. aureus* phagocytosis in mDC differentiated in the presence of COX-2 KO ASCs or parental ASCs. Data representative of three independent experiments. ASC, adipose-derived mesenchymal stem cell; CD, cluster of differentiation; Exp, experiment; KO, knock-out; mDC, mature dendritic cell; *S. aureus*, *Staphylococcus aureus*.

**Additional file 4: Suppl. Figure 4.** OLINK analysis of the secretome from Mphs and mDCs. The table gathers the NPX of all the inflammation panel targets in the secretome of the different Mph populations and mDCs. Statistically significant (*p* < 0.05) fold changes in NPX compared with M0 non-polarized Mphs are highlighted in green (upregulation) or red (downregulation) (*n* = 4). N/D targets in all populations are excluded: ARTN, BDNF, βNGF, CCL25, CD6, CX3CL1, FGF19, FGF23, GDNF, IL-15RA, IL-17a, IL-17c, IL-2, IL-20, IL-20RA, IL-22RA1, IL-24, IL-2RB, IL-33, IL-5, LIFR, NRTN, NT3, SIRT2, SLAMF1, TRANCE, and TSLP. IFNγ, IL-4, and IL-13 are not shown because they were added exogenously in M1 and M2 populations, respectively, and were N/D in the other populations. 4E-BP1, eukaryotic translation initiation factor 4E-binding protein 1; ADA, adenosine deaminase; ARTN, artemin; BDNF, brain-derived neurotrophic factor; CASP, caspase; CCL, C-C motif chemokine; CD, cluster of differentiation; CDCP, CUB domain-containing protein; CSF, macrophage colony-stimulating factor; CST5, cystatin D; CX3CL1, fractalkine; CXCL, C-C-C motif chemokine; DNER, delta and notch-like epidermal growth factor-related receptor; EN.RAGE, protein S100-A12; FGF, fibroblast growth factor; Flt3L, fms-related tyrosine kinase 3 ligand; GDNF, glial cell line-derived neurotrophic factor; HGF, hepatocyte growth factor; IFN, interferon; IL, interleukin; LAP, latency-associated peptide; LIF, leukemia inhibitory factor; LIFR, leukemia inhibitory factor receptor; MCP, monocyte chemotactic protein; mDC, Mature dendritic cell; MIP, macrophage inflammatory protein; MMP, matrix metalloproteinase; Mph, Macrophage; N/D, non-detected (under low-limit of detection); OPG, osteoprotegerin; OSM, oncostatin-M; PD-L1, programmed death ligand 1; NGF, nerve growth factor; NPX, Normalized Protein eXpression; NRTN, neurturin; NT-3, neurotrophin-3; SCF, stem cell factor; SIRT, SIR2-like protein 2; SLAMF1, signaling lymphocyte activation molecule; ST1A1, sulfotransferase 1A1; STAMBP, STAM binding protein; TGF, transforming growth factor; TNF, tumor necrosis factor; TNFRSF, tumor necrosis factor receptor superfamily member; TNFSF, tumor necrosis factor ligand superfamily member; TRAIL, tumor necrosis factor-related apoptosis-inducing ligand; TRANCE, tumor necrosis factor-related activation-induced cytokine; TSLP, thymic stromal lymphopoietin; TWEAK, tumor necrosis factor ligand superfamily member 12; uPA, urokinase-type plasminogen activator; VEGF, vascular endothelial growth factor.

## Data Availability

The datasets used and/or analyzed during the current study are available from the corresponding author on reasonable request.

## Abbreviations

7-AAD: 7-Aminoactinomycin D
ASC: Adipose-derived mesenchymal stem cell
CCL: C-C motif chemokine
CCR: C-C motif chemokine receptor
CD: Cluster of differentiation
CDCP: CUB domain-containing protein
CM: Conditioned media
CO_2_: Carbon dioxide
COX: Cyclooxygenase
crRNA: CRISPR RNA
CX3CR: CX3C chemokine receptor
CXCL: C-X-C motif chemokine
CXCR: C-X-C chemokine receptor
DC: Dendritic cell
DMEMc: Complete Dulbecco’s modified Eagle’s medium
*E. coli*: *Escherichia coli*
EN.RAGE: Extracellular newly identified receptor for advanced glycation end-products binding protein
Exp: Experiment
FBS: Fetal bovine serum
FSC-A: Forward scatter area
FSC-H: Forward scatter height
GDNF: Glial cell line-derived neurotrophic factor
GM-CSF: Granulocyte-macrophage colony-stimulating factor
HLA: Human leukocyte antigen
iDC: Immature dendritic cell
IFN: Interferon
IL: Interleukin
KO: Knockout
LIF: Leukemia inhibitory factor
LOD: Limit of detection
LPS: Lipopolysaccharide
MCP: Monocyte chemotactic protein
M-CSF: Macrophage colony-stimulating factor
mDC: Mature dendritic cell
MIP: Macrophage inflammatory protein
Mph: Macrophage
MSC: Mesenchymal stem cell
OPG: Osteoprotegerin
P: Parental
PBMC: Peripheral blood mononuclear cell
PBS: Phosphate-buffered saline
PGE2: Prostaglandin E2
qPCR: Quantitative polymerase chain reaction
RPMI: Roswell Park Memorial Institute
RPMIc: Complete Roswell Park Memorial Institute
*S. aureus*: *Staphylococcus aureus*
SSC: Side scatter
TGF: Transforming growth factor
Th1: Type 1 T helper
Th2: Type 2 T helper
TLR: Toll-like receptor
TNF: Tumor necrosis factor
TNFSF: Tumor necrosis factor ligand superfamily member
tracrRNA: Trans-activating CRISPR RNA
