# Supplementary figures and images for: Human adipose mesenchymal stem cells modulate myeloid cells toward an anti-inflammatory and reparative phenotype: role of IL-6 and PGE2
Source: Stem Cell Res Ther. 2020 Nov 2;11:462. doi: 10.1186/s13287-020-01975-2 (PMC7607855; doi:10.1186/s13287-020-01975-2)

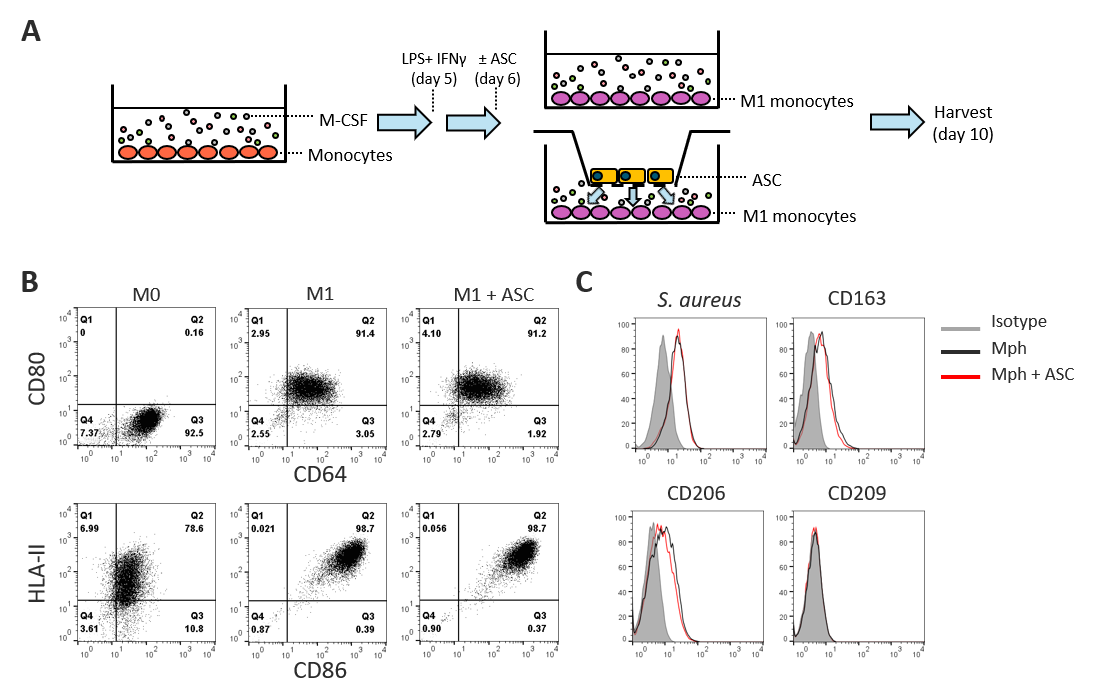

Supplement: Supplementary file 1 — Additional file 1: Suppl. Figure 1. Reversion of M1 pro-inflammatory macrophage phenotype by ASC co-culture. A: Diagram shows the experimental settings of M1 Mph reversion assay using ASCs; B: Graphs show CD80/CD64 and HLA-II/CD86 surface expression of M1 Mph in the presence or absence of ASCs; C: Histograms showing the phagocytosis of S. aureus pHrodo™ particles and CD163, CD206 and CD209 expression by M1 Mphs in the presence or absence of ASCs, measured by flow cytometry. Data representative of two independent experiments. ASC, adipose-derived mesenchymal stem cell; CD, cluster of differentiation; IFN, interferon; LPS, lipopolysaccharide; M-CSF, Macrophage Colony-Stimulating Factor; Mph, macrophage; S. aureus, Staphylococcus aureus. [file 13287_2020_1975_MOESM1_ESM.tif]

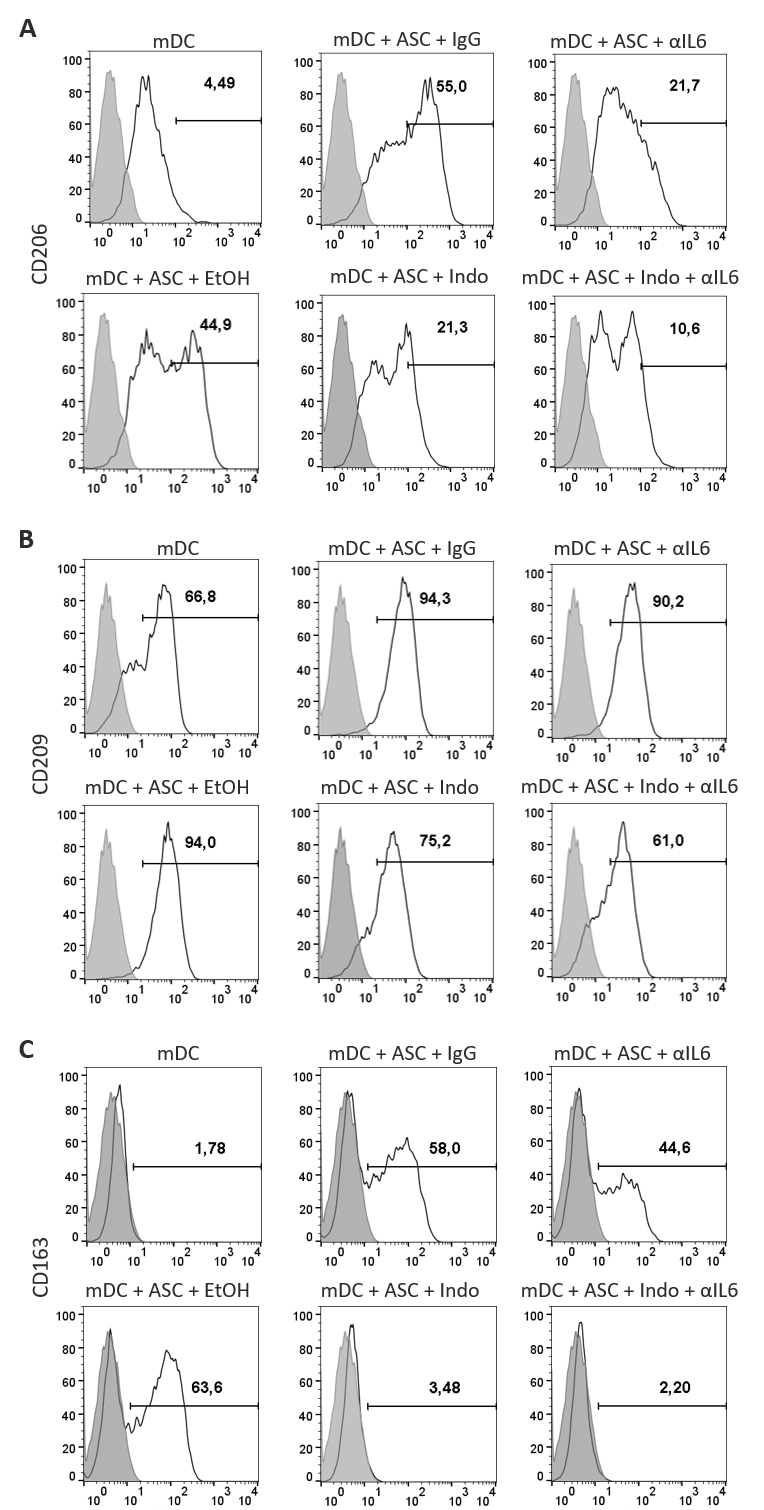

Supplement: Supplementary file 2 — Additional file 2: Suppl. Figure 2. Surface expression levels of phagocytic receptors in ASC-educated mDCs modulated by IL-6 and PGE2 inhibitors. Histograms show the surface expression of A: CD206; B: CD209; and C: CD163, in monocyte-derived mDCs in the presence or absence of ASCs and modulated by IL-6 or PGE2 inhibitors, measured by flow cytometry. IgG1 was the negative control for αIL-6, and ethanol was added as indomethacin carrier (n = 3). ASC, adipose-derived mesenchymal stem cell; CD, cluster of differentiation; EtOH, ethanol; IgG, immunoglobulin G; Indo, indomethacin; IL, interleukin; mDC, mature dendritic cell; PGE2, prostaglandin E2. [file 13287_2020_1975_MOESM2_ESM.tif]

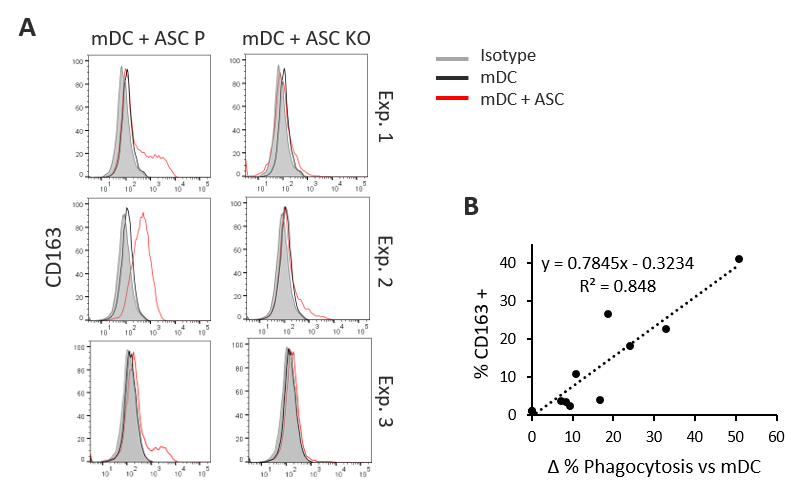

Supplement: Supplementary file 3 — Additional file 3: Suppl. Figure 3. CD163 surface expression of mDC differentiated in the presence of COX-2 KO ASCs or parental ASCs. A: Histograms show the surface expression of CD163 of mDC differentiated in the presence of COX-2 KO ASCs or parental ASCs; B: Correlation between CD163 expression and increase in percentage of S. aureus phagocytosis in mDC differentiated in the presence of COX-2 KO ASCs or parental ASCs. Data representative of three independent experiments. ASC, adipose-derived mesenchymal stem cell; CD, cluster of differentiation; Exp, experiment; KO, knock-out; mDC, mature dendritic cell; S. aureus, Staphylococcus aureus. [file 13287_2020_1975_MOESM3_ESM.tif]

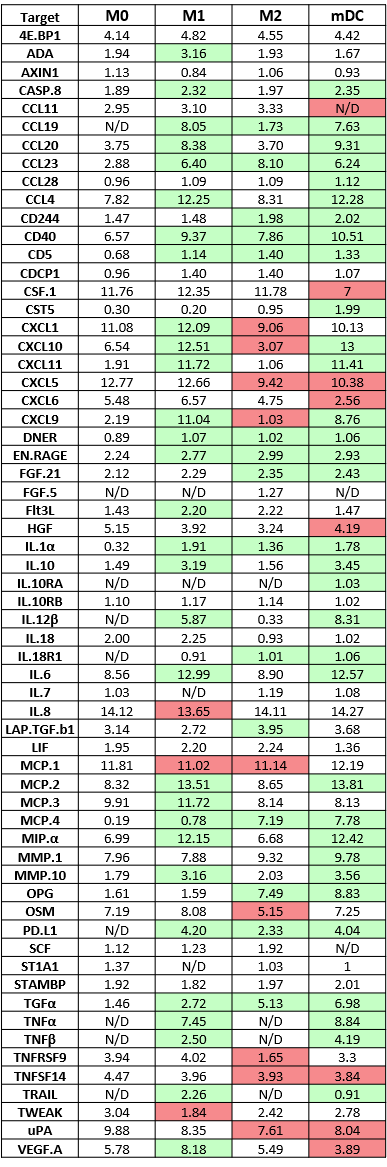

Supplement: Supplementary file 4 — Additional file 4: Suppl. Figure 4. OLINK analysis of the secretome from Mphs and mDCs. The table gathers the NPX of all the inflammation panel targets in the secretome of the different Mph populations and mDCs. Statistically significant (p < 0.05) fold changes in NPX compared with M0 non-polarized Mphs are highlighted in green (upregulation) or red (downregulation) (n = 4). N/D targets in all populations are excluded: ARTN, BDNF, βNGF, CCL25, CD6, CX3CL1, FGF19, FGF23, GDNF, IL-15RA, IL-17a, IL-17c, IL-2, IL-20, IL-20RA, IL-22RA1, IL-24, IL-2RB, IL-33, IL-5, LIFR, NRTN, NT3, SIRT2, SLAMF1, TRANCE, and TSLP. IFNγ, IL-4, and IL-13 are not shown because they were added exogenously in M1 and M2 populations, respectively, and were N/D in the other populations. 4E-BP1, eukaryotic translation initiation factor 4E-binding protein 1; ADA, adenosine deaminase; ARTN, artemin; BDNF, brain-derived neurotrophic factor; CASP, caspase; CCL, C-C motif chemokine; CD, cluster of differentiation; CDCP, CUB domain-containing protein; CSF, macrophage colony-stimulating factor; CST5, cystatin D; CX3CL1, fractalkine; CXCL, C-C-C motif chemokine; DNER, delta and notch-like epidermal growth factor-related receptor; EN.RAGE, protein S100-A12; FGF, fibroblast growth factor; Flt3L, fms-related tyrosine kinase 3 ligand; GDNF, glial cell line-derived neurotrophic factor; HGF, hepatocyte growth factor; IFN, interferon; IL, interleukin; LAP, latency-associated peptide; LIF, leukemia inhibitory factor; LIFR, leukemia inhibitory factor receptor; MCP, monocyte chemotactic protein; mDC, Mature dendritic cell; MIP, macrophage inflammatory protein; MMP, matrix metalloproteinase; Mph, Macrophage; N/D, non-detected (under low-limit of detection); OPG, osteoprotegerin; OSM, oncostatin-M; PD-L1, programmed death ligand 1; NGF, nerve growth factor; NPX, Normalized Protein eXpression; NRTN, neurturin; NT-3, neurotrophin-3; SCF, stem cell factor; SIRT, SIR2-like protein 2; SLAMF1, signaling lymphocyte activati [file 13287_2020_1975_MOESM4_ESM.tif]
